# Role of Non-Coding RNAs in the Etiology of Bladder Cancer

**DOI:** 10.3390/genes8110339

**Published:** 2017-11-22

**Authors:** Caterina Gulìa, Stefano Baldassarra, Fabrizio Signore, Giuliano Rigon, Valerio Pizzuti, Marco Gaffi, Vito Briganti, Alessandro Porrello, Roberto Piergentili

**Affiliations:** 1 Department of Gynecology, Obstetrics and Urology, Policlinico Umberto I, Sapienza University of Rome, 00161 Rome, Italy; 85cate@live.it (C.G.); stefano.baldassarra@hotmail.it (S.B.); 2 Department of Obstetrics and Gynaecology, Misericordia Hospital, 58100 Grosseto, Italy; fabri64@me.com; 3 Department of Obstetrics and Gynaecology, Azienda Ospedaliera San Camillo-Forlanini, 00152 Rome, Italy; darra80@hotmail.com; 4 Department of Urology, Misericordia Hospital, 58100 Grosseto, Italy; vpizzuti@gmail.com; 5 Pediatric Surgery and Urology Unit, Azienda Ospedaliera San Camillo-Forlanini, 00152 Rome, Italy; marco.gaffi59@gmail.com (M.G.); vito.briganti@fastwebnet.it (V.B.); 6 Lineberger Comprehensive Cancer Center, University of North Carolina at Chapel Hill, Chapel Hill, NC 27599, USA; 7 Institute of Molecular Biology and Pathology, Italian National Research Council (CNR-IBPM), 00185 Rome, Italy

**Keywords:** *TP53*, *FGFR3*, microRNA, small non-coding RNA, long non-coding RNA, epigenetics

## Abstract

According to data of the International Agency for Research on Cancer and the World Health Organization (Cancer Incidence in Five Continents, GLOBOCAN, and the World Health Organization Mortality), bladder is among the top ten body locations of cancer globally, with the highest incidence rates reported in Southern and Western Europe, North America, Northern Africa and Western Asia. Males (M) are more vulnerable to this disease than females (F), despite ample frequency variations in different countries, with a M:F ratio of 4.1:1 for incidence and 3.6:1 for mortality, worldwide. For a long time, bladder cancer was genetically classified through mutations of two genes, fibroblast growth factor receptor 3 (*FGFR3*, for low-grade, non-invasive papillary tumors) and tumor protein P53 (TP53, for high-grade, muscle-invasive tumors). However, more recently scientists have shown that this disease is far more complex, since genes directly involved are more than 150; so far, it has been described that altered gene expression (up- or down-regulation) may be present for up to 500 coding sequences in low-grade and up to 2300 in high-grade tumors. Non-coding RNAs are essential to explain, at least partially, this ample dysregulation. In this review, we summarize the present knowledge about long and short non-coding RNAs that have been linked to bladder cancer etiology.

## 1. Introduction

As stated by the US National Cancer Institute (NCI) [1], bladder cancer (BC) is the sixth most frequent type of cancer in the Western world, especially in the USA, in which it represents 4.7% of all new cancer cases. According to statistical projections, in 2017, there will be 79,030 new cases of BC and an estimated 16,870 individuals will die because of it. BC is most commonly diagnosed among people aged 75–84, with a median age of 73 years, meaning that the rising life expectancy of the older population itself is increasing the number of BC cases, turning them into a bigger problem for the healthcare [2]. BC is more frequently diagnosed in men than in women and its five-year survival rate, based on data from “Surveillance, Epidemiology, and End Results Program” (SEER), is estimated at 77.3% [1]. BC is the ninth leading cause of cancer death in the USA with 4.4 deaths per 100,000 men and women per year (time frame: 2010–2014), according to SEER data.

## 2. Classification and Etiology of BC

There are two main types of BC, according to its histopathology: about 90% of them begin in the urothelium (the transitional epithelium that lines much of the urinary tract including the renal pelvis, the ureters, the bladder and parts of the urethra) and are classified as transitional cell carcinoma or urothelial cell carcinoma; additionally, approximately 5% of BC are squamous cell carcinoma and less than 2% are adenocarcinoma [3]. BC can also be classified considering its invasion of the *muscularis propria* in the bladder wall: at the time of diagnosis, 75–80% cases of BC are classified as non-muscle-invasive bladder cancer (NMIBC), consisting of the pathologic stages Ta (papillary), T1 (invading into the *lamina propria*), and carcinoma in situ (CIS) [4]. The remaining cases are characterized by a variable grade of infiltration of the bladder wall and are known as muscle-invading BC (MIBC), which are a more serious condition: in fact, while patients with NMIBC can often be safely managed with transurethral resection of the tumor (with or without intravesical chemotherapy or immunotherapy), neoadjuvant chemotherapy followed by radical cystectomy (RC) with bilateral pelvic lymph-node dissection (PLND) is the standard of care for patients with MIBC [5].

Many studies focused on possible etiologies of BC, demonstrating that both genetic and epigenetic pathways are correlated with the development of this disease, and that BC is also strongly influenced by environmental factors [6]. In past studies, BC was commonly linked to two main genetic pathways, one involving *FGFR*3 (fibroblast growth factor receptor 3,) mutations, often associated with low-grade tumors and favorable prognosis, and one characterized by *TP53* (tumor protein P53) mutations, mainly identified in advanced tumors with poor prognosis [7]. Currently, numerous other genetic causes have been discovered, concerning several categories of oncogenes and tumor suppressor genes whose altered activities promote carcinogenesis: (i) genes controlling apoptosis [8] such as *Caspase-3* [9], *FAS* (Fas cell surface death receptor) [10], *BCL-2* (B-Cell CLL/Lymphoma 2) [11] and *Survivin* [12]; (ii) genes controlling angiogenesis such as vascular endothelial growth factors (VEGFs) [13], basic fibroblast growth factor and thrombospondin 1 [14]; and (iii) genes controlling signaling and cell–cell interactions as uroplakins [15] and *RAS* (Rat sarcoma viral oncogene homolog) [16]. Many genes involved in other cellular functions seem to play a role in BC development [17] and, for this reason, it may be useful to analyze as many of them as possible to define prognosis and treatments.

Among the most important risk factors, there are cigarette smoking, occupational exposures (especially to aromatic amines), water arsenic, *Schistosoma haematobium* infestation, and external beam radiation therapy (EBRT) for urogenital malignancies, which increases the rate of secondary bladder malignancies [17,18]. Cigarette smokers have approximately threefold higher hazard of urinary tract cancer than non-smokers [19]. It has been discovered that cigarette smoke extract exposure induces morphological changes of human BC T24 cells, with enhanced cell migration and invasion, reduced epithelial marker expression and increased mesenchymal marker expression, resulting in the activation of the ERK1/2 (Extracellular Signal-regulated Kinase 1 and 2) pathway as well as of the activator protein 1 (AP-1) [20]. About 20% of BC cases is occupationally related, a much larger percentage compared to other types of cancer which have, in some cases, a work-dependent etiology (4%) [21]. Workers in leather tanneries have a higher hazard to develop BC, likely because of their exposure to chemicals contained in the leather dust [22]. Evidence of higher BC risk was also shown in dye workers, rubber workers, painters, truck drivers and aluminum workers [23]. Many studies focused on the personal use of hair dyes, which contain several chemicals: *p*-phenylenediamine and aminophenyl, for example, have been suggested as possible carcinogens or mutagens. These molecules, when used over a long period of time and in the presence of specific genetic polymorphisms, may increase BC risk [24], sometimes up to 22–50% vs. non-use [25]. It has been established the fundamental role that aromatic amines, such as 2-naphthylamine, 4-aminobiphenyl and benzidine, play as occupational risk factors associated with BC [26]. Another important hazard is the exposure to arsenic in drinking water: an arsenic concentration of 10 μg/liter may increase by 40–100% the risk of bladder and kidney cancers [27]. There are also some world regions in which BC is linked to environmental factors that almost make it an endemic disease: for example, in the Middle East and parts of Africa (especially Egypt), carcinomas of the urinary tract are the most common malignancies, due to *S. haematobium* infections [28].

## 3. Epigenetics and BC

Many scholars have been asked about the meaning of the term ‘epigenetic’: hence different definitions and points of view emerged. Overall, ‘epigenetics’ includes all the biochemical processes that alter gene activity without changing the DNA sequence and that may lead to inheritable modifications [29]. Therefore, epigenetic changes can alter the way a cell interacts with its genetic information, without altering the DNA sequence, at the physiological level.

The hunt for gene variants or mutations that can explain the personal susceptibility to diseases, from autism to cancer to Alzheimer’s disease, has been mostly inconclusive. For this reason, epigenetics may be the key to find a more complete pathogenetic explanation for diseases that do not have clear-cut genetic causes [30]. There are many biochemical processes, which determine epigenetic changes. However, most studies focused on two of them: DNA methylation and histone modifications [31]. DNA methylation (the addition or removal of a methyl group) has been the focus of much research ranging from a developmental and imprinting perspective to its contribution to diseases such as cancer [32]. Aberrant histone modifications, or the dysregulated activity of the enzymes that catalyze these modifications, may affect the genome integrity and chromosome segregation, leading to the possible development of many maladies, including cancer [33]. Even though these phenomena have been known for decades, studies investigating the impact of epigenetic changes on human diseases have only recently intensified.

Remarkably, epigenetic changes mediated by non-coding RNAs (ncRNA) are of extreme importance for understanding the neoplastic transformation of the urothelium. Indeed, there is not only a direct relationship between activity and mutations of these molecules, intended as members of a complex epigenetic system [34], and BC formation [35], but also a known association between some chemicals and changes of ncRNA structures [36]. In particular, benzo(a)pyrene (produced during coal gasification, aluminum smelting and tobacco smoking, in all cases as a result of incomplete combustion), 2-nitrofluorene (a by-product of combustion) and 4-nitrosomorpholine (a contaminant in rubber products) can potentially impair the function of some ncRNA and, probably, induce BC [37].

## 4. Non-Coding RNAs: An Overview

Modern studies and technologies allowed pointing out that only a small portion (about 10–15%) of the mammalian genome is functional [38], and only 3% consists of protein-coding genes [39]. The greatest part of the human genome, which does not encode for proteins, is known as ‘non-coding DNA’: in fact, it does not lead to the biosynthesis of specific proteins. However, additional studies have demonstrated that non-coding DNA holds a very important role in many other biochemical processes. Indeed, it carries the information that is required to synthesize specific RNA molecules, which are not translated into proteins (ncRNA). Among others, transfer RNAs (tRNA) and ribosomal RNAs (rRNA) represent the most abundant ncRNA molecules, confirming the central role played by this huge part of the human genome. ncRNA, other than rRNA and tRNA, are usually categorized based on their length: there are long non-coding RNAs (lncRNA), which are longer than 200 nucleotides [40] and small non-coding RNA, which are usually 20–25 nucleotides long and are further subdivided according to their function and/or role (Table 1 and References therein).

Over the years, many roles have been attributed to ncRNA. It has been proposed that ncRNA play a crucial role in maintaining genomic stability, which is essential for cell survival and prevent tumorigenesis [51], by finely tuning DNA expression [52] and forming complexes with other molecules (lncRNA, microRNA (miR) and proteins) to maintain the physiologic homeostasis [53].

## 5. Long Non-Coding RNA

**Figure 1:**
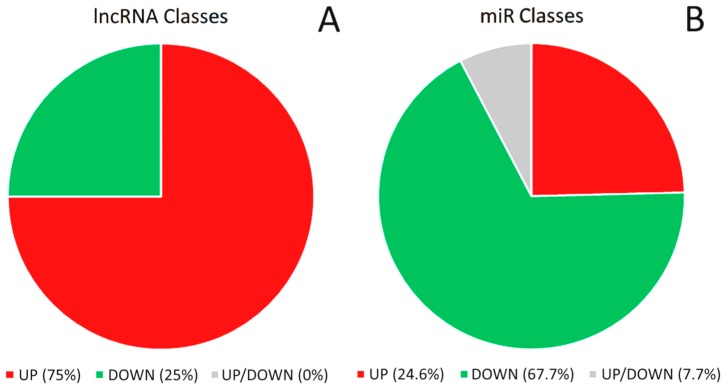
Classification of long non-coding RNA (lncRNA) and micro-RNA (miR) involved in bladder cancer (BC) etiology based on their regulation. (**A**) lncRNA classes according to their levels of expression in BC vs. normal urothelium. lncRNA are divided into two classes: (i) lncRNA that are up-regulated (in red; 24/32); and (ii) lncRNA that are down-regulated (in green; 8/32). To date, BC-related lncRNA have been univocally described as up- or downregulated. (**B**) miR classes according to their levels of expression in BC vs. normal urothelium. miR are split into three classes: (i) miR that are upregulated (in red; 16/65); (ii) miR that are downregulated (in green; 44/65); and (iii) miR that have been described both as up- and downregulated and, as such, cannot be univocally classified using the available data (in grey; 5/65). Notably, while 75% of the BC-related lncRNA are upregulated (i.e., they act as oncogenes), more than 70% of these miR are potentially downregulated (considering all downregulated and half of the non-univocally classified miR), i.e., act as tumor suppressors.

Long Non-coding RNA are involved in many important biochemical processes such as the major pathways of cell growth, proliferation, differentiation and survival. Consequently, they are considered essential regulators of the genetic information and for this reason, in the last years, several studies focused on their activities. Their deregulation can promote tumor formation, progression and metastasis in bladder tissues, showing that they play a crucial role in the carcinogenesis of the urothelium [54]. lncRNA can sustain proliferative signaling, allow evading growth suppression, promote apoptosis resistance, support replicative immortality, activate invasion and metastasis, and induce angiogenesis [55]. These findings are a great step forward for understanding cellular pathways and defining the prognosis of a large variety of tumors; indeed, many studies aim to translate this evidence into actionable clinical steps, for example using specific lncRNA as biomarkers or therapeutic targets [41]. Transcribed ultraconserved region (T-UCR) RNAs are a specific class of lncRNA possibly involved in tumorigenesis; notably, one out of the approximately 480 T-UCR identified so far has a recognized role in BC formation [56]. The lncRNA that we have collected (Table 2) and commented have been described as having a role in BC and were retrieved from PubMed and the database ‘Lnc2Cancer’ available at http://www.bio-bigdata.com/lnc2cancer/home.jsp [57]. Their role as oncogenes or tumor suppressor is summarized in Figure 1A.

### 5.1. UCA1

Urothelial cancer-associated 1 (UCA1) is encoded in a locus that maps on chromosome 19p13.12 and is 1413 bases long [60]. It belongs to the family of oncogenic lncRNA involved in BC progression because of its role in cell cycle regulation. It is believed to promote BC indirectly acting on the PI3K-AKT (phosphatidylinositide 3-kinase—Ak thymoma) pathway through the CREB (cAMP response element-binding protein) protein deregulation [60]. Under hypoxic conditions, the upregulation of UCA1 promotes cell proliferation, migration, and invasion; furthermore, it inhibits apoptosis [61]. It has been shown that in ovarian cancer UCA1 interacts with the microRNA miR485-5p, inhibiting its function [62]. In this system, the knockdown of UCA1 or the overexpression of miR485-5p reduce mRNA and protein levels of the target matrix metalloprotease MMP14, which is involved in pathologic invasion and metastasis. A similar pathway has been demonstrated in BC as well, in an article showing that miR485-5p is a tumor suppressor [63]. Further studies are needed to verify that UCA1 and miR485-5p interact also in the bladder. Several studies have investigated the reliability of the UCA1 presence in the urine as a biomarker for urothelial cancer, demonstrating its great diagnostic value in BC (specificity: 91.8%; sensitivity: 80.9%) [64,65]; however, its role in the follow-up of recurring tumors remains limited [66]. Moreover, growing subsequent evidence suggests that the aberrant overexpression of UCA1 is associated with high risk of poor outcome or clinicopathological features in several types of cancer, including BC [67]. UCA1 activity is strictly related to aggressive BC phenotypes; in fact, UCA1 can promote the transdifferentiation of epithelial cells into motile mesenchymal cells, a process known as epithelial–mesenchymal transition (EMT) [68]. This feature can be explained by the effect that UCA1 produces through the hsa-miR-145–ZEB1/2–FSCN1 (*Homo sapiens* miR-145—Zinc Finger E-box binding homeobox 1 and 2—fascin actin-bundling protein 1) pathway. It has been demonstrated that hsa-miR-145 inhibits BC cell migration and invasion [69]; consequently, the overexpression of UCA1 is linked to marked repression of hsa-miR-145, and vice versa, resulting in a stimulus that may make cancer more aggressive. In addition, UCA1 induces EMT and increases the migratory and invasive abilities of BC cells also by upregulating the expression levels of the zinc finger E-box binding homeobox 1 and 2 (ZEB1 and ZEB2) [70].

### 5.2. MALAT1

Metastasis-associated lung adenocarcinoma transcript 1 (MALAT1), also known as nuclear-enriched abundant transcript 2 (NEAT2), is encoded in a locus that maps on chromosome 11q13.1 and its length is around 7 Kb [71]. Interestingly, the 3′ end of the main transcript contains a conserved tRNA-like sequence that is cleaved off and processed to generate a short tRNA-like ncRNA called mascRNA (MALAT1-associated small cytoplasmic RNA; 61nt long); consequently, the post-transcriptional processing of MALAT1 produces two ncRNAs from a single, original transcript [72]. Both transcripts are widely expressed in all human tissues analyzed so far. MALAT1 is often associated with multiple physiological processes, such as alternative splicing, nuclear organization, and epigenetic modulation of gene expression. However, this lncRNA is also involved in pathological processes, ranging from diabetes complications to different types of cancer [73]. The role of MALAT1 in tumorigenesis has been deeply investigated; its overexpression has been associated with the promotion of malignancy, while its knockdown is linked to the inhibition of cell proliferation and invasion in different cancer types [74]. However, the implications of MALAT1 in carcinogenesis have not yet been fully clarified because of the controversial results that emerged from numerous studies. The overexpression of MALAT1 is generally associated with poor prognosis in patients with various types of cancer, even though the mechanisms underlying this relationship remain unclear [75]. The upregulation of MALAT1 promotes cell migration in BC by inducing EMT; its downregulation results in a decrease of this process [76]. These findings suggest an important role for MALAT1 in regulating metastasis in BC patients and its possible therapeutic use as a target molecule. Since cancer patients expressing high levels of this lncRNA have a poorer clinical outcome, MALAT1 can be considered a potential prognostic biomarker for various cancers, including BC [77].

### 5.3. H19

*H19* is a locus mapping on chromosome 11p15.5 (inside a genomic region that is approximately 6 Kb long), which encodes for a lncRNA and is also known as ASM (because of its expression in rats’ Adult Skeletal Muscle) or BWS (since its aberrant expression can be involved in the Beckwith-Wiedemann syndrome). H19 is an imprinted transcript, expressed exclusively from the maternal allele, and is only found in mammalian genomes [78]. It is expressed during fetal development, in particular in mesoderm- and endoderm-derived tissues [79], and its expression ends at birth; in adults, it is found almost exclusively in a number of cancers, including BC [80,81]. As for the bladder, Ariel et al. demonstrated that H19 is abundantly expressed in the fetal bladder mucosa and in the carcinoma of the urinary bladder [82]. H19 is a potent oncogene: its aberrant expression is associated with BC tumorigenesis, metastasis, and poor prognosis, since it is one of the most upregulated lncRNA in cancerous cells compared with adjacent noncancerous tissue [83]. The search for H19-positive urinary cells has become a highly sensitive screening test for urothelial cancer; in fact, H19 has been detected in the urine of 90.5% of patients and only in 25.9% of controls [84]. The molecular organization and mechanisms of action of this ncRNA are quite complex and not fully understood. The primary transcript of *H19* also acts as the primary transcript for miR-675, a 23nt long microRNA harbored between nucleotides 1014 and 1036 of the main transcript [85]. In addition, the H19/IGF2-imprinting control region, which is located just upstream of *H19*, coordinates the expression of both *H19* and *IGF2* (insulin like growth factor 2), the latter being expressed exclusively from the paternal allele [86]. *H19* knockout causes loss of *IGF2* imprinting, suggesting a control mechanism at the transcriptional level [87,88]; in addition, both sense and antisense transcripts from the *H19* locus bind the PRC2 (polycomb repressive complex 2) chromatin modification complex in mouse embryonic stem cells [89]. However, H19 also binds the IGF2 mRNA binding-protein (IMP) family members that, in turn, regulate IGF2 translation, thus suggesting for H19 also a post-transcriptional regulation of target genes [90]. One of the pathological mechanisms involved in the H19 activity is linked to the expression of miR-675: notably, miR-675 levels were remarkably increased in BC tissues, where this miR is able to inhibit the activation of the tumor suppressor TP53, resulting in the abnormal proliferation of BC cells and increased cancer growth [91]. miR-675 also downregulates the tumor suppressor retinoblastoma (RB) protein in human colorectal cancer [92]. However, it is likely that H19 has also functions that are independent of miR-675, since it harbors secondary structures that are widely conserved and may act as a stable docking platform for a regulatory ribonucleoprotein (RNP) composed of the 3′ half of the H19 transcript and of up to four IMP1 molecules [93].

### 5.4. TUG1

Taurine Upregulated Gene 1 (*TUG1*) maps on chromosome 22q12.2, in a locus of approximately 10 Kb. Three splice variants of *TUG1* were identified in mouse, while in man there are four possible splice variants, whose cDNA lengths are 3.3, 5.9, 6.4, and 9.7 kb, respectively [94]. Human TUG1 is ubiquitously expressed in tissues and cells and localizes in nuclear and cytoplasmic foci in human fetal foot fibroblasts [95]. TUG1 is another lncRNA whose aberrant overexpression is commonly linked to urothelial carcinoma of the bladder, as well as to B-cell malignancies, esophageal squamous cell carcinoma, hepatocellular carcinoma and osteosarcoma [96]. TUG1 knockdown suppresses proliferation and promotes apoptosis of BC cells, by inhibiting the activation of the Wnt/β-catenin (wingless-type MMTV integration site/beta-catenin) pathway and by affecting Zinc finger E-box-binding homeobox 2 (ZEB2) expression [97]. A research demonstrated the oncogenic role and association with worse overall survival of this lncRNA, especially in high-grade muscle-invasive BC: in this study, TUG1 silencing in vitro led to 34% decrease in cancer cell proliferation and 23% reduction in the migration capacity of cancer cells [98]. The role of TUG1 in high-grade BC may be related to its interaction with TP53, a well-known marker of these tumors [17]. Indeed, *TUG1* expression is induced by TP53 in response to DNA damage [99], and the artificial depletion of TUG1 via small interfering RNA (siRNA) results in the upregulation of genes involved in cell cycle regulation in both fetal lung and foot fibroblasts [95]. Further studies are needed to better understand the importance of TUG1, though its potential roles as biomarker and therapeutic target in bladder urothelial carcinoma seem to be well established.

### 5.5. MEG3

There are also cases in which the reduced expression of a certain lncRNA can lead to several pathologies, and cancer is one of them. One example is represented by Maternally Expressed Gene 3 (MEG3) lncRNA, whose cytogenetic band is 14q32.2 (a site that putatively contains a tumor suppressor gene involved in the pathogenesis of meningiomas) and which has a complex organization, being ca. 1.6 kb long in humans with a number of splice isoforms and evidence of retained introns that create longer transcripts [100]. In addition, in this case, there is an example of an imprinted, maternally expressed locus [101] that is part of a larger cluster of imprinted genes, named *DLK1-DIO3* (delta like non-canonical notch ligand 1—iodothyronine deiodinase 3); like the *IGF2/H19* domain, the *MEG3/GTL2/DLK1* locus has areas of differential methylation-hypermethylation on the paternal and hypomethylation on the maternal allele [102]. *MEG3* expression is highly regulated temporally and spatially in mouse as well as in man. In humans, it is expressed in the adult brain [103] and normal pituitary gland (but not in pituitary adenomas) [104]; it is upregulated in the *nucleus accumbens* of heroin abusers [105] and downregulated in the caudate nucleus of Huntington’s disease patients [106]. MEG3 localization may be either nuclear or cytoplasmic; when it is nuclear, it has been found associated with chromatin [107]. The downregulation of *MEG3* is commonly associated with the progression of different types of cancer; in fact, MEG3 has the strong ability to inhibit the proliferation of several malignant human carcinoma cell lines [104]. It has been demonstrated that this anti-oncogene contributes to the activation of *TP53*, one of the most important tumor suppressor genes, probably through secondary structural motifs [108]. Furthermore, MEG3 is able to inhibit cell proliferation even in the absence of TP53 [109]; in addition, it can also control gene expression at imprinted loci through the recruitment of the PRC2 complex [89]. Experiments aimed at elucidating its role in cellular growth further demonstrated that its ectopic expression inhibits growth and stimulates *TP53* expression [108]. On the other hand, *MEG3* knock-out promotes the expression of VEGF (vascular endothelial growth factor) signaling pathway genes in the brain, suggesting that MEG3 function as tumor suppressor may in part be due to angiogenesis inhibition [110]. As in other tissues, also in BC MEG3 levels are lower than normal controls. This lncRNA is able to suppress autophagy activation, while downregulated MEG3 activates autophagy and increases cell proliferation in BC cell lines [111]. The downregulation of *MEG3* is based on the epigenetic silencing of the 14q32 imprinted gene cluster [112]. All biochemical processes in which MEG3 is involved make it an excellent marker for BC prognosis: circulating MEG3 status in tumors may be useful also for selecting patients who are most likely to benefit from adjuvant therapy, which is used to reduce the risk of cancer recurrence [113].

### 5.6. MIR31HG

MIR31HG (also known as LOC554202) is another example of lncRNA whose inactivation promotes the progression of BC, as well as of other types of cancer. *MIR31HG* maps on chromosome 9p21.3 [114] and its locus spans more than 106 kb, although the final transcript is only 2246 bp long; exon 1 contains a CpG island, and intron 1 harbors miR31 [115]. Interestingly, the regulation of this lncRNA depends on the cancer type. The CpG island is responsible for gene expression control through DNA methylation in triple-negative breast cancer (TNBC) cell lines [115]. It has also been shown that MIR31HG is upregulated in breast cancer tissues compared with normal tissues and that higher MIR31HG expression positively correlates with tumor size and staging. On the contrary, its knockdown via siRNA reduces proliferation, migration, and invasion in breast cancer cell lines, with accumulation of cells in the G0/G1 phase and loss of cells in S phase, and at the same time increases apoptosis [114]. The relationship between decreased expression of MIR31HG and development of BC suggests its role as tumor-suppressor in bladder, and its expression levels in BC patients are negatively correlated with advanced TNM (tumor-node-metastasis) stage [116]. Thus, MIR31HG is a new candidate biomarker for patients with BC, although the molecular mechanism by which it is regulated in the bladder has still to be elucidated.

### 5.7. Linc-UBC1

Long intergenic non-coding RNA upregulated in bladder cancer 1 (linc-UBC1, approved by Human Genome Organization (HUGO) Gene Nomenclature Committee (HGNC) symbol: BLACAT1) is a lncRNA that is overexpressed in BC and was found to be over-expressed in about 60% of invasive BC tissue specimens; it was correlated with lymph node metastasis and poor survival [117]. The gene maps at position 1q32.1 in a locus longer than 20 Kb, but its final transcript is only 3 Kb long (a single exon); this transcript localizes mainly inside the nucleus [117]. RNA immunoprecipitation experiments revealed that linc-UBC1 interacts with two components of the PRC2 complex; thus, this RNA probably functions, at least partially, by modulating the histone methylation and chromatin structure, and consequently influencing gene expression [117].

### 5.8. LOC572558

LOC572558 (cytogenetic band of its gene: 9q13) has been recently identified in BC specimens [118] and is one of the most deregulated lncRNA in BC; it can be considered an important tumor suppressor, which regulates the p53 signaling pathway in BC by dephosphorylating AKT and MDM2 (mouse double minute 2 homolog) and phosphorylating TP53 protein. Available data show that it is able to inhibit cell proliferation and motility; in fact, it can induce S phase arrest of the cell cycle and promote apoptosis [119].

### 5.9. PANDAR

Promoter of CDKN1A antisense DNA damage-activated RNA (PANDAR) is a lncRNA that regulates the expression of genes involved in the apoptotic response to DNA damage [120]; the coding gene maps at position 6p21.2 and its transcript is approximately 1.5 Kb long. PANDAR is significantly upregulated in BC tissues, compared with adjacent non-tumoral tissues; moreover, its high levels were correlated with higher histological grade and advanced TNM stage. It was shown that, after silencing PANDAR, cell proliferation and migration are inhibited, while apoptosis is induced [121]. Hung et al. suggested that the DNA damage induces TP53-mediated transcription at the locus containing both CDKN1A and PANDAR; this transcription mediates cell cycle arrest and inhibits NFYA (nuclear transcription factor Y subunit alpha, a transcription factor involved in the activation of pro-apoptotic genes) [120]. A recent report also shows the role of PANDAR in the stabilization of TP53 protein without influencing the TP53 mRNA stability [122].

### 5.10. GHET1

Gastric carcinoma high expressed transcript 1 (GHET1), is a recently identified lncRNA, originally isolated in patients affected by gastric carcinoma, where it is upregulated; the high expression levels of this RNA are directly correlated with tumor size, tumor invasion and poor survival [123]. The gene location is 7q36.1. GHET1 is upregulated in BC as well [124]; indeed, Li et al. showed its involvement in the proliferation and invasion of BC cells in vitro and in the EMT of BC cell lines, and that its upregulation is directly related with tumor status and size, but not with other variables such as age or gender. They conclude that GHET1 contributes to the tumor progression and may be used as a novel diagnostic BC marker.

### 5.11. ncRAN

Another recently discovered, upregulated lncRNA involved in BC formation is ncRAN (non-coding RNA expressed in aggressive neuroblastoma) [125], a long ncRNA originally isolated in patients affected by aggressive neuroblastoma [126]; its gene maps at position 17q25.1 and has two similarly sized transcripts, whose lengths are 2.1 and 2.2 Kb, respectively. Both ncRAN transcripts are significantly more expressed in invasive bladder tumor cell lines than in superficial tumor cell lines, where they promote cell proliferation, migration, and invasion [125]. It is currently unknown if the two isoforms differ in some way with respect to cancer, including BC cases. Similarly, the targets of ncRAN are currently unknown. Therefore, ncRAN, despite being a promising BC biomarker, is still considered only a ‘potential’ oncogene and further research is needed to clarify its biological role [125].

### 5.12. GAS5

Growth arrest specific 5 (*GAS5*) is a gene mapping at position 1q25.1. The *GAS5* primary transcript (technically, a lncRNA) contains 12 exons in both man and mouse. However, these exons are poorly conserved, and the specific search of putative polypeptides derived by alternative splicing has been so far inconclusive [127,128]. Instead, its introns are highly conserved, and their analysis, comparing human and mouse *GAS5* sequences, revealed that they contain several small nucleolar RNA (snoRNA) (that are small non-coding RNAs (sncRNA)) in the same number and order, i.e., 11 introns containing nine conserved snoRNA arranged in the same way [127]. In 2013, Liu et al. demonstrated that, in most BC samples, *GAS5* transcription is significantly downregulated [129], as in breast cancer [130], allowing classifying this gene as a tumor suppressor. Instead, *GAS5* overexpression in proliferating cells is sufficient to stop the cell division [129]. Interestingly, in the same paper it is reported that Cyclin-dependent kinase 6 (CDK6) is specifically associated with GAS5, thus permitting assigning this transcript to at least one target gene and a specific role in the BC formation.

### 5.13. ANRIL

Antisense non-coding RNA in the INK4 locus (ANRIL) is encoded by a gene that maps on chromosome 9p21.3; two splicing forms are known, of 2.7 and 3.8 Kb. It was originally discovered by searching EST (expressed sequence tag) databases in a region of chromosome 9, which is frequently deleted in the melanoma-neural system tumor (NST) syndrome [131]. ANRIL seems to be ubiquitously expressed in human tissues (in 20 tissues examined), at the same level and always together with CDKN2A/INK4/ARF (cyclin-dependent kinase inhibitor 2A—inhibitor of CDK4—alternate reading frame), whose genes map next to that of ANRIL and might be regulated by the same factors [131]. ANRIL is dysregulated in several cancers, including BC; in bladder, it regulates cell proliferation and apoptosis through the intrinsic pathway, since its knockdown corresponds to decreased expression of *BCL-2*, increased expressions of *BAX* (BCL2 Associated X), cytoplasmic *cytochrome c* and *SMAC* (second mitochondria-derived activator of caspases), and cleavage of Caspase-9, Caspase-3 and PARP (poly ADP ribose polymerase) [132].

### 5.14. HIF1A-AS2

Hypoxia inducible factor 1 alpha antisense RNA-2 (HIF1A-AS2) was originally discovered in samples of human renal cancer; its gene maps on chromosome 14q23.2 and, reportedly, produces a AU-rich transcript of 1577 nucleotides [133]. The 3’ 882 nucleotides of HIF1A-AS2 are completely complementary to the 3’-UTR of HIF1A, which is transcribed in the opposite orientation, and indeed its upregulation corresponds to a downregulation of HIF1A in a lymphocyte cell line under hypoxia stress [133]. These results, which are cell line specific, were subsequently further validated upon camptothecin treatment [134,135]. Recently, HIF1A-AS2 upregulation was found also in BC samples, where its expression levels are positively associated with advanced clinical pathologic grade and TNM stage [136].

### 5.15. HOTAIR and HOXD-AS1

HOX (homeobox) transcript antisense RNA (HOTAIR/HOXAS) belongs to a ncRNAs family, identified in human fibroblast, which has roughly 200 HOX [137]. This RNA is transcribed from a locus mapping on chromosome 12q13.13; it was described that the gene encodes a 2158 nt long ncRNA whose function is to provide a modular scaffold for multiple histone modification complexes [138]. Interestingly, its pattern of expression is position-dependent as it happens for the HOX genes, i.e., HOTAIR is more expressed towards the posterior and distal sites in the adult human body [137]. Its depletion through RNA interference causes the upregulation of several HOX genes, as well as several HOX ncRNA, and loss of the histone-3 trimethylation on lys27 over the HOXD locus; the contemporary loss of SUZ12 (suppressor of zeste 12) in the same chromosome domain supports the hypothesis that HOTAIR might regulate the polycomb repressive complex-2 (PRC2) localization and HOXD silencing [137]. Dysregulation of HOTAIR has been linked to several cancer types, including malignancies of the breast and of epithelial types, and is associated with more aggressive tumor behaviors and metastasis formation [139]. Its connection with BC has been demonstrated recently: in particular, its upregulation is a hallmark of recurrence in stage Ta/T1 [140] probably because of its modulation of the cancer epigenome [141]. This possibility is further supported by the fact that HOTAIR contributes to change the balance of histone modification between H3K4me3 and H3K27me3 on the miR-205 promoter, thus causing the silencing of miR-205, which in turn has a role in the inhibition of cell proliferation, migration and invasion by direct targeting of the *cyclin J* (*CCNJ*) gene, a regulator of cell cycle progression [142].

HOXD cluster antisense RNA 1 (HOXD-AS1, also known as HOXD antisense growth-associated long non-coding RNA, HAGLR) belongs to the HOXD cluster on chromosome 2 (at position 2q31.1) and has been recently associated to BC formation and progression; in particular, Li et al. demonstrated that its synthetic tetracycline-controllable shRNA targeting is sufficient to inhibit the progression of BC, although it is unclear how this happens [143].

### 5.16. MDC1-AS1

Mediator of DNA damage checkpoint protein 1 (MDC1) is a regulator of the intra-S phase and G2/M cell cycle checkpoints whose role is recruiting DNA repair proteins to the site of damage. It is involved in determining cell survival fate in association with tumor suppressor protein TP53. Mediator of DNA damage checkpoint protein 1 antisense RNA 1 (MDC1-AS1, encoded at location 6p21.33) is the antisense transcript of the same gene. Xue et al. found that the expression levels of MDC1-AS1 and MDC1 are both downregulated in BC and there is an inhibitory role of this antisense RNA on the malignant cell behavior of EJ and T24 BC cell lines [144]. Interestingly, the same study demonstrated that the over-expression of MDC1-AS1 promotes the upregulation of the MDC1 coding gene both at RNA and protein levels, suggesting that MDC1-AS1 has an inhibitory role on BC cells proliferation through its upregulation of the tumor suppressor gene *MDC1*. While this subject is still under investigation, the easiest explanation found so far is that, in some way, this antisense RNA can stabilize the MDC1 mRNA.

### 5.17. PCAT-1

Prostate Cancer Associated Transcript 1 (PCAT-1) was originally identified as a biomarker of prostate cancer [145], but subsequently it was also found to be involved in the progression of colorectal cancer [146]. This ncRNA transcript has two exons: exon 1 contains a retroviral long terminal repeat (LTR) sequence derived from LTR78B, while exon two contains sequences from the HSMAR1 mariner family transposase that, in turn, internally hosts an AluY repeat element. *PCAT-1* maps in the 8q24 locus, which is frequently amplified in prostate cancer; however, it was shown that PCAT-1 upregulation is not dependent on genomic amplification [145]. PCAT-1 is upregulated in BC tissues compared to healthy controls, indicating its oncogenic role; moreover, its depletion by shRNA (small hairpin RNA) treatment in T24 and 5637 BC cell lines causes cell growth arrest and induction of apoptosis, suggesting that it is a possible BC therapeutic candidate [147].

### 5.18. PVT1

Plasmacytoma variant translocation (*Pvt1*) oncogene (*PVT1*) maps on chromosome 8q24.21, a locus that is frequently involved in t(2;8) translocations that are present in some human Burkitt lymphomas. PVT1 RNA and MYC protein expression correlate in several primary human tumors, and a direct relationship between copy number of PVT1 and MYC copy-increase has been found in more than 98% of human cancers. On the contrary, the PVT1 downregulation in the MYC-driven colon cancer cell line HCT116 reduced its tumorigenic potential, indicating a direct role of PVT1 in controlling MYC abundance. [148]; instead, the PVT1 overexpression is independent of MYC, at least in some cancers [149]. Similar studies were performed on BC samples as well, demonstrating that: (i) in BC, PVT1 is upregulated; indeed, in BC cells this lncRNA is highly correlated with histological grade and TNM stage. (ii) PVT1 silencing by shRNA inhibits the BC progression and promotes apoptosis [150]. Since MYC protein is refractory to small-molecule inhibition, the dependence of high MYC protein levels on PVT1 lncRNA suggests a promising way to therapeutically target this protein in MYC-positive cancers.

### 5.19. SChLAP1

The gene of the SWI/SNF complex antagonist associated with prostate cancer 1 (SChLAP1) maps on chromosome 2q31.3 and produces at least seven transcripts; however, more than 90% of these transcripts only belong to four splicing variants of 1.7, 1.4, 1.3 and 1.1 kb. It was originally isolated in aggressive prostate cancer, where its expression is in direct relationship with the metastasis formation [151]. In the same study, it was reported that the SChLAP1 knockdown in prostate cancer cell lines altered their gene expression profile in a way that was contrary to that of the SWI/SNF chromatin-modifying complex. The authors supported this finding by showing that this ncRNA coprecipitated with SMARCB1 (SWI/SNF related, matrix associated, actin dependent regulator of chromatin, subfamily B, member 1), a subunit of the SWI/SNF complex and concluded that SChLAP1 antagonizes the SWI/SNF function by attenuating the binding to its genomic targets. Similar results were recently obtained in BC; however, the mechanisms of action of SChLAP1 still have to be elucidated [152].

### 5.20. SPRY4-IT1

SPRY4 intronic transcript 1 (SPRY4-IT1) is harbored inside the second intron of the *SPRY4* (sprouty RTK signaling antagonist 4) gene (an inhibitor of the MAPK kinase signaling pathway); this gene maps on chromosome 5q31.3. SPRY4-IT1, reportedly, is an unspliced, polyadenylated lncRNA with 708 nucleotides. It is not conserved among primates and, in normal human tissues, is most highly expressed in placenta, kidney, and lung [153]. In melanoma patient samples, its overexpression has been linked to cell proliferation, invasion and mobility; as expected, its knockdown causes opposite cellular effects, thus allowing classifying it as a potential oncogene [153]. Analogous results were obtained in other cellular systems, such as renal cancer, esophageal squamous cell carcinoma and trophoblast cells. Zhao et al. described a similar scenario in urothelial carcinoma of the bladder, where SPRY4-IT1 is overexpressed and significantly linked to histological grade, tumor stage, lymph node metastasis and reduced overall survival [154].

### 5.21. ZEB2-AS1

ZEB2 (zinc finger E-box binding homeobox 2, whose gene maps at position 2q22.3) is a transcriptional repressor of E-cadherin; it is upregulated after SNAI1-induced EMT. However, SNAI1 does not affect the synthesis of ZEB2 mRNA, but prevents the processing of a large intron located in its 5′-UTR region. ZEB2 Antisense RNA 1 (ZEB2-AS1, also named ZEB2 natural antisense transcript, ZEB2NAT) is the lncRNA contained inside the *ZEB2* locus and harbors an internal ribosome entry site necessary for the expression of Zeb2. The expression of this antisense transcript overlapping the 5′ splice site in the intron prevents the splicing of the ZEB2 5′-UTR and increases the quantity of the ZEB2 protein; this outcome downregulates E-cadherin both at the mRNA and protein levels [155]. In BC patient samples, the tumor invasiveness is driven by the TGFβ1 signaling pathway that promotes the EMT through cancer-associated fibroblasts (a major component of the cancer stroma); in this system, TGFβ1 is overexpressed in the presence of an upregulated ZEB2-AS1 (together with ZEB2) [156].

### 5.22. T-UCR 8+

Approximately 480 sequences in the human genome show a 100% identity with orthologous sequences in mice and rats, indicating that they went through a very strong negative selection for 300–400 million years; these regions are called ‘ultraconserved’ and some of them are transcribed as ncRNA [157]. In many cases, the function of these ncRNA is still to be explained; some are likely involved in splicing [158], others map next to transcriptional regulators or developmental genes, suggesting a related role for them [159], others are probably connected with cell proliferation, since they have copy number abnormalities in cancer tissues [160]. One of them has been linked to BC, i.e., ultraconserved RNA 8+ (uc.8+), located within the intron 1 of *CASZ1* (castor zinc finger 1, encoding a zinc-finger transcription factor), although it is expressed independently of *CASZ1* [56]. uc.8+ is the most upregulated T-UCR in BC tissues, but its expression is lower than in pericancerous bladder tissues. uc.8+ downregulation significantly reduces cancer cell invasion, migration and proliferation. Data available strongly suggest that uc.8+ is a natural trap for miR-596; as a result, the action of uc.8+ would be to deplete the intracellular availability of this miR and induce the upregulation of its targets, including MMP9 (matrix metalloprotease 9, involved in the degradation of extracellular matrix molecules), thus promoting cell proliferation and migration [56].

### 5.23. NEAT1

Nuclear enriched abundant transcript 1 (NEAT1) is a polyadenylated, unspliced ncRNA, which is abundantly transcribed in several cancers (bladder, lung, and breast) and promotes their development and progression; the same locus also encodes a short noncoding RNA, TncRNA (trophoblast-derived non-coding RNA), that originates from the 3’ end of NEAT1 and is exclusively expressed in trophoblasts [71]. *NEAT1* maps in the locus 11q13.1, less than 70 kb apart from *MALAT1*. Recently, Qian et al. demonstrated that its action in BC is performed through miR-101 tumor suppressor direct targeting that, successively, regulates EZH2 (enhancer of zeste homolog 2) function [161], while Ke et al. reported that NEAT1 is required for BC cell survival through FUS (fused in sarcoma) and miR-548 [162]. In an interesting recent article, it was described that: (i) NEAT1 is up-regulated in BC tissues and cell lines; and (ii) the knock-down of this lncRNA inhibits cell proliferation, suppresses cell migration and induces apoptosis in 5637, T24 and SW780 human BC cell lines [163].

### 5.24. Other lncRNA Deregulated in Bladder Cancer

For some BC-related lncRNA only preliminary evidence is available and/or data are clearly incomplete, especially as for mechanisms of action and target identification. In this section, we briefly mention cases that fall into this category.

Apoptosis-associated transcript in bladder cancer (AATBC, whose cytogenetic band is 21q22.3) is overexpressed in BC and positively correlates with tumor grade and pT stage; its inhibition causes cell proliferation arrest in G1 mediated by cyclin D1, CDK4, p18 and phosphorylated RB, as well as apoptosis induction through the intrinsic pathway [164].

Additionally, the search of new lncRNA is currently exploiting the ‘omics’ sciences, by analyzing the genome, the transcriptome, chromatin immunoprecipitation samples, etc. In this way, typically thousands of molecules are analyzed with a single experiment and bioinformatics analyses can be performed using the experimental output. Zhu et al. performed a microarray screening of lncRNAs in four pairs of human BC and matched normal tissues [118]. They could identify 110 lncRNA that are significantly (≥8 times) dysregulated in BC, and went on to validate by quantitative PCR (qPCR) four possible candidates (TNXA, CTA-134P22.2, CTC-276P9.1 and KRT19P3; Table 2), for which additional analyses are required to establish their role in this pathology. A similar approach has been used by Chen et al., who found out that lncRNA-n336928 is upregulated in BC, being positively correlated with BC grade and stage and negatively with patient survival [165]. Finally, Zhang et al. identified lncRNA-UNMIBC (long non-coding RNA-Up-regulated in non-muscle invasive bladder cancer) through a microarray analysis of lncRNA expression in BC samples. This molecule is upregulated in non-muscle invasive BC and the authors, through RNA and chromatin immunoprecipitation, showed that it is physically associated with EZH2 and SUZ12 (which are core components of PRC2), leading to an altered histone H3 lysine 27 methylation status of the target genes [166].

We conclude this paragraph by citing the articles of Peter et al. [167] and Wang et al. [168] as two examples of the growing complexity of the research in the field of lncRNA and BC. Both groups performed a genome-wide analysis of lncRNA differentially expressed in BC cells vs. normal urothelium. In the first article, the expression of 17,112 lncRNA was evaluated through microarray analysis; in this way, the authors identified 32 molecules potentially important in BC progression. They focused on AB074278, which was picked for the following features: “(i) associated with disease progression; (ii) upregulated in all urothelial cancer phenotypes […] (iii) had low predicted protein-coding scores (thus likely to be a ncRNA); (iv) worse outcomes with high expression (thus a potential oncogenic role); (v) also upregulated in urothelial cancer; and (vi) appeared of particular interest as it was intronic (sense direction) to a protein coding host gene (sense to TANC2; as were most validated ncRNAs in GENCODE) also upregulated in urothelial cancer (thus potentially regulated by the lncRNA […])” [167]. Notably, while AB074278 met these requirements, still there is no certainty in defining it as a ‘genuine’ upregulated lncRNA directly involved in the BC etiology and the identified interactions need further validation, exemplifying the difficulties of the scientists working in this field. As for the second paper [168], the authors examined the expression of 33,045 lncRNA through microarray analysis, revealing 3419 lncRNA differentially expressed in BC, with fold changes between 2 and 43 (1905 upregulated and 1514 downregulated). In addition, in this case, the identified lncRNA candidates need further, specific validation to understand their potential diagnostic and prognostic value in BC.

## 6. Small Non-Coding RNA

The definition of ‘small non-coding RNA’ includes many different, highly heterogeneous molecules (Table 1). Among them, a pivotal role in BC etiology is played by a specific class of RNAs, the microRNAs (also abbreviated in the literature as miRNA or miR). The study of miR is considered a great opportunity to better understand some pathological mechanisms, especially those linked to carcinogenesis. miR play important regulatory roles in animals and plants by targeting mRNAs for cleavage or translational repression [169]. Interestingly, these non-coding RNAs partly explain the (now obsolete) concept of ‘junk DNA’. After the completion of the human genome sequencing, it was evident that only 2% of the human DNA encodes functional proteins; 50% to 75% of the genome is transcribed, and 98% of the transcripts are not translated into proteins [170]. Non-coding RNAs are transcribed from approximately 70% of the genomic regions that used to be considered ‘junk DNA’ [171], i.e., meaningless DNA regions (from a genetic point of view). A significant number of miR are arranged in groups having an approximate length of 10 kb; this kind of organization is usually referred as ‘miR cluster’. The miR of a cluster are usually co-expressed, because they are under the control of a common promoter, and even share some target genes [172]. Moreover, miR also have some level of functional redundancy, as shown by sequence comparison, which likely is a useful backup system to protect normal cells from the malignant transformation [173].

As mentioned above, miR are usually ca. 22 nt long and their typical hairpin structure displays complementary pairing with their target mRNA [174]; therefore, it has been proven that a single sncRNA is able to target several mRNAs, usually by binding one of its ends or, in some cases, one of its internal sequences [175]. This field of molecular biology is extensively investigated because of its multiple implications for medicine (particularly medical oncology). In fact, the presence of miR not only in tissues but also in extracellular fluids (blood, urine and cerebrospinal fluid) suggests that these molecules can be used as informative biomarkers for the early diagnosis of diseases and as diagnostic tools in general [176]. miR can be roughly divided in two broad groups, according to the biological features and pathological mechanisms in which they are involved. miR which, upon upregulation, can be linked to a specific cancer are considered oncogenes; instead, miR whose downregulation takes part in carcinogenesis, are considered tumor suppressors. Indeed, miR activity in controlling gene expression, in cancer as well as in various other important diseases, makes them ideal candidates for therapeutic applications. Notably, miR selective modulation through antisense inhibition (for upregulated miR) or replacement (i.e., restoring a downregulated miR function by providing an external source of miR, through a vector overexpressing the targeted miR or transfecting a double-stranded miR) could significantly affect the prognosis of several diseases [177].

### 6.1. Micro-RNA and Bladder Cancer

The involvement of different miR in the pathogenesis of BC is the subject of ongoing research. The goal is to clarify and exploit the use of miR as BC biomarkers, prognostic factors, and therapeutic targets [178], as well as in other pathological conditions linked to their altered expression. The list of miR that target genes involved in BC formation and development is already quite long. In 2011, Zhu et al. [179] reported the results of an NGS analysis that allowed identifying 226 differentially expressed miR in BC (182 upregulated and 44 downregulated); of these, 104 upregulated and 20 downregulated sequences were specific for BC, while the remaining were in common with other genitourinary malignancies (i.e., kidney and testicular cancer). In addition, Chen et al. [180] found tens of altered miR in BC—33 upregulated and 41 downregulated—when compared with normal bladder epithelium. Zobolotneva et al. described, through a systematic analysis of scientific reports, 95 differentially expressed miR, of which 48 were upregulated, 35 downregulated and 12 not clearly classifiable [181]. In another study [182], the number of downregulated miR was 60, with 17 new potential miR identified. A recent meta-analysis on 473 papers published between 2009 and 2016 shows that at least 118 miR were identified multiple times in BC samples, or were detected in at least two out of three biological samples (tissue, blood, urine); of these, 111 miR were found in BC clinical specimens, with 57 downregulated or silenced, 51 overexpressed and the remaining cases contradictory [183]. Most likely these lists will become longer in the next few years, because of the great research effort that this field is producing. Indeed, through these investigations scientists hope to better understand the epigenetic mechanisms, which contribute to bladder carcinogenesis and to plan effective and targeted therapies. However, in most of these studies, only the deregulation of miR is considered, without any clue about the miR role in BC etiology. A recent meta-analysis performed by searching the PubMed and Google Scholar databases for publications ranging between 1990 and 2016 allowed identifying at least 35 miR specifically associated with different pathways of cellular dedifferentiation, proliferation, and progression of BC as well as of other cancers [184]. In this review, we have focused only on miR with an established role in urothelium neoplastic transformation by means of at least one identified target gene (Table 3) and have discussed only a few of them as prominent examples. However, since the choices made by different Authors to characterize miR target genes are based on very heterogeneous criteria, we have decided to collect all of them in this table as “proposed” targets. The role of these miR as oncogenes or tumor suppressor, according to their expression, is summarized in Figure 1B.

### 6.2. The FGFR3 Pathway

Two main genetic pathways predispose to bladder carcinogenesis, for which the altered expression of miR is very important. Some miR, such as miR-99a, miR-100, miR-101, and miR-145, target the FGFR3 pathway, determining gain-of-function mutations, which are mostly detected in NMIBC; on the other hand, miR such as miR-21 and miR-373 cause loss-of-function mutations in the TP53 pathway, which are commonly found in MIBC [188].

The fibroblast growth factor (FGF) family of transmembrane tyrosine kinase receptors mediates proliferation in response to FGF stimulation and has been implicated in the pathogenesis of urothelial carcinoma: fibroblast growth factor receptor 3 (FGFR3) is frequently mutated or overexpressed in NMIBC [189]. For this reason, FGFR3 represents a useful BC biomarker with low malignant potential [190]. The evaluation of FGFR3 mutational status became relevant for human diseases when specific point mutations were discovered in various autosomal dominant human skeletal diseases, including achondroplasia, hypochondroplasia, thanatophoric dysplasia I and II, and severe achondroplasia with developmental delay and *acanthosis nigrans* (SADDAN) (reviewed in [191]). It was later shown that those mutations were also present in human bladder and cervix carcinomas [192]. Further investigations showed that *FGFR3* mutations occurred at a much higher rate in BC than in other tumor types (e.g., cervix, multiple myeloma, gastrointestinal tract, prostate) [193]. Several miR are involved in the FGFR3 pathway linked to BC formation and development (Table 3). For example, low levels of miR-100 were correlated with low-grade, non-invasive bladder urothelial cancer, due to the upregulation of FGFR3, meaning that, under physiological conditions, miR-100 acts as a tumor suppressor [194]. Similar activities are carried out also by miR-31, whose decreased expression has been described in the urothelial carcinoma of the bladder: miR-31 may contribute to the BC progression and is associated with unfavorable prognosis, overall and progression-free survival [195]. In addition, the downregulation of miR-99a leads to the upregulation of *FGFR3*, yet it is most commonly linked to low-grade tumors and better outcome [196]. miR-34a negatively regulates the cell cycle by reducing CCND1 (cyclin D1) and CDK4 (cyclin dependent kinase 4) levels; its downregulation promotes a more aggressive behavior of FGFR3 in urothelial carcinoma cases [197]. Other miR regulate this pathway, by acting either directly or indirectly on FGFR3 [181], as illustrated in Table 3; it is likely that the list of miR involved in the control of this gene will become longer in the future.

### 6.3. The TP53 Pathway

The other major genetic trigger, which can predispose to the formation and development of BC is represented by the loss of function of Tumor Protein p53 (TP53). TP53 is a transcription factor essential for the prevention of cancer formation in most human tissues. This gene encodes several protein isoforms, whose study has had a profound impact on our understanding of TP53 activity [198]. TP53 is one of the most important tumor suppressor genes: it provides essential functions in the cellular response to diverse stresses, safeguards the maintenance of genomic integrity and is a potent inducer of apoptosis and senescence when expressed in tumor cells [199]. TP53 can induce cell cycle arrest, DNA repair and, eventually, senescence and/or apoptosis; its mutations have been reported to occur in almost every type of cancer at rates varying between 10% (e.g., in hematopoietic malignancies) and almost 100% (e.g., in ovarian high-grade serous carcinoma) [200]. The frequency of molecular changes of this gene is substantially higher in MIBC (43.4%) than in NMIBC (8.2%), and a strong association exists between TP53 mutations and high tumor stage [201]. A computational analysis of the TP53 amino acid sequence has shown that this protein has remarkable similarities with prions [202] and experimental research has confirmed that amyloid formation can explain the negative dominance and loss of function of cancer-associated mutant TP53 [203,204]. Additionally, the TP53 pathway can be impaired by the action of several miR molecules, which may also induce loss-of-function (Table 3). For example, miR-205 has an ambivalent behavior as for the neoplastic transformation: indeed, it has many physiological and crucial roles, but its aberrant expression has been linked to the formation of many malignancies. miR-205 can act either as a tumor suppressive or an oncogenic miR, by regulating different cellular pathways such as those of cell survival, apoptosis, angiogenesis and metastasis, depending on its target genes [205]. Indeed, miR-205 is potentially able to target at the same time TP53, PTEN (Phosphatase and tensin homolog), c-erbB3 (a member of the epidermal growth factor receptor family of receptor tyrosine kinases), CDC42 (a protein involved in the cell cycle regulation), and YES (Yamaguchi sarcoma viral oncogene homolog) proto-oncogene 1 [206]. Therefore, miR-205 is possibly involved in many biochemical processes, including carcinogenesis. miR-205 may act as a diagnostic and prognostic BC marker, since it has been discovered that a mucosa with a normal appearance may have molecular changes (that precede phenotypic changes) in the form of varied expression of this molecule, besides other miR, such as miR-129 and miR-200a [184]. miR-21 targets many genes that can be involved in the formation and development of cancer, including TP53 [207], PTEN, TIMP3 (tissue inhibitor of metalloproteinase 3) (both inhibitors of the matrix metalloproteinases), BCL-2 (regulator of apoptosis) [208], and many others; its overexpression is linked to loss of function of TP53, which is most commonly observed in high-grade MIBC [209]. The overexpression of miR-373 is also found in BC, due to its many functions that promote carcinogenesis, cell invasion and metastasis [210]. Interestingly, miR-373 may act either as an oncogene or a tumor suppressor [210] and indirectly impairs TP53 function. Finally, some proteins regulated by miR, such as TP53 [211], the double-stranded RNA-specific endoribonuclease DROSHA and PTEN [212], are in turn able to regulate miR expression (Table 3); thus, some components of the miR maturation machinery are themselves under miR control in BC.

### 6.4. Other Molecular Pathways Causing miR-Mediated BC Formation

It is generally accepted that *FGFR*3 and *TP*53 alterations characterize alternative genetic pathways in the pathogenesis of urothelial cell carcinoma and are hallmarks of specific BC types: *FGFR3* mutations are observed in 59% and *TP53* overexpression in 25% of primary BC. These alterations are almost mutually exclusive, since they overlap in only 5.7% of all tumors [213]; in addition, 15% of BC are normal with respect to both genes. However, it is well known that a single mutation is not sufficient to induce the neoplastic transformation, and that several tens of genes and different biochemical pathways are altered in BC samples at the same time, most of which have a direct role in cell cycle regulation, apoptosis, angiogenesis, cell–cell interaction, DNA repair, chromatin structure, gene expression, etc. [17]. As shown in Table 3, there are several miR that do not target neither FGFR3 nor TP53, directly or indirectly [181], yet are deregulated in BC specimens; these data strongly suggest that the deregulation of several genes at the same time is required for the neoplastic transformation of the normal urothelium.

### 6.5. New Approaches for Assessing the miR-Mediated BC Formation

A peculiar point of view about the role played by miR in the formation and development of BC is that, in some cases, it is more informative to evaluate the ratio between two miR than to measure the total level of any single miR. For example, the miR-21:miR-205 expression ratio has the ability to distinguish between invasive and non-invasive bladder tumors with high sensitivity and specificity, and with the potential to identify superficial lesions that have a high risk of progression [206]. In another report, the ratio between miR-126 and miR-152 in the urine allowed the BC detection with a sensitivity of 72% and specificity of 82% [214]. Finally, a recent report shows that the miR-182:miR-100 ratio in BC specimens may fulfill several diagnostic requirements. In BC, miR-182 is usually upregulated, while miR-100 is downregulated. This ratio is associated with pT-stage, histological grade, BC recurrence and presence of carcinoma in situ; when this ratio is high, it is significantly correlated with shortened BC survival [215]. As a consequence, the miR-182:miR-100 ratio is a novel, non-invasive, promising biomarker for the diagnosis and survival assessments of BC patients.

## 7. Other ncRNA Involved in BC Etiology

As previously described, the epigenetic etiology of BC mostly involves two main categories of ncRNA, long ncRNA and miR. We conclude our review by mentioning a few other non-coding RNA molecules that have been linked to this pathology.

### 7.1. Y RNA

Y RNA were originally identified as the RNA component of soluble ribonucleoproteins (RNPs) named Ro RNPs. Four human Y RNAs have been identified: Y1, Y3, Y4 and Y5 (Y2 is a truncated form of Y1). They have two recognized functions: repressors of Ro60 and other Ro proteins and initiation factors for the DNA replication [216]. In 2008 Christov et al. [217] found that two human Y RNA, namely hY1 and hY3, are significantly overexpressed in BC and several other tumors, and their RNA interference (RNAi)-mediated degradation results in a significant cytostatic (but not cytotoxic) effect in cell lines, probably by inducing a significant inhibition of chromosomal DNA replication in cultured human cells. These data, coupled with the fact that short fragments of Y RNAs have recently been identified as abundant components in the blood and tissues of humans and other mammals, make this class of ncRNA a valuable potential tool not only for diagnostic purposes, but also as a possible therapeutic target [218].

### 7.2. circRNA

Circular RNA (circRNA) are a class of RNA molecules that are covalently closed in a loop at the 3′ and 5′ ends. This peculiarity makes them more resistant than linear RNA to exonuclease-mediated degradation; indeed, their half-life is estimated to be approximately 48 h or more, while linear mRNA last on average 10 h [43]. circRNA can be generated via splicing from exons (exonic circRNA) or introns (intronic circRNA); these two classes, as well as a third class, i.e., retained-intron circular RNA, have distinct biogenesis patterns [219]. To date, their cellular role is still largely unknown. For some of them, it has been shown a role as miR sponges, i.e., they are able to bind miR without being degraded, thus preserving the mRNA target of the miR themselves [43]. In other cases, it has been proposed a role for circRNA, especially exonic ones, in the regulation of transcription or translation by direct targeting of the mRNA they come from, in such a way that their potential purposes are served through their mechanism of formation, rather than as final biological molecules [43]. In addition, their role as scaffold for the formation of RNA binding protein complexes cannot be ruled out [43]. Overall, circRNA have been described as potential decoys that interfere with the destination and function of their molecular counterparts and that have relevance in several cancer types [220]. Studies of circRNA in BC are still at the beginning, yet they are very promising. Zhong et al. demonstrated that at least six circRNA are highly expressed in human BC samples; in particular, circTCF25 can upregulate the levels of CDK6 by acting as a RNA sponge for miR-103a-3p and miR-107, thus promoting both cell proliferation and metastasis formation [221]. Similarly, circRNA-MYLK (encoded inside the myosin light chain kinase gene, MYLK) and circRNA-CTDP1 (mapping inside the gene encoding the subunit 1 of the phosphatase of the C-terminal domain of RNA polymerase II subunit A, CTDP1) can competitively bind miRNA-29a-3p and increase the expression of its target genes (DNMT3B, DNA methyltransferase 3 Beta; VEGFA, vascular endothelial growth factor A; HAS3, hyaluronan synthase 3; ITGB1, integrin subunit Beta 1), while circRNA-PC (inside the pyruvate carboxylase gene) is a competitor of miR-185-3p, a miR that targets ADD1 (adducin 1) and BAP1 (breast cancer 1 associated Protein 1) [83,222].

## 8. Conclusions

In the last years, significant steps have been taken towards the understanding of the role of epigenetics in human diseases in general, and cancer in particular. Far from being ‘junk DNA’, most of the human genome that does not code for proteins is important for cell homeostasis. Part of this genome is transcribed into RNA molecules of variable length, whose role in chromatin structure, gene expression, cell cycle control and (co-)regulation of many important biological pathways is currently investigated. RNA molecules with an altered expression in BC are many and, likely, their list will become larger in the future; indeed, important tools to identify and quantify these molecules are already available. We believe that the biggest challenges in this field will be precisely characterizing the functions of these RNAs inside the cells and taking advantage of this new knowledge to improve the health of BC patients.

## Figures and Tables

**Table 1:** Main non-coding RNA (ncRNA) classes, listed according to increasing length (in nucleotides (nt)).

Name	Acronym	Length (nt)	Notes	Refs
Long non-coding RNAs	lncRNA	>200	Non-protein coding transcripts; heterogeneous class of RNAs	[41]
Transcribed ultraconserved region	T-UCR	≈50–570	Frequently located at fragile sites and cancer-associated genomic regions; possibly regulated by miR	[42]
Circular RNA	circRNA	≈100–1600	Covalently closed RNA rings; some have coding functions; potential gene regulators and miR “traps”	[43]
Small interfering RNA	siRNA	20–25	Double-stranded RNAs similar to miR, operating through RNA interference (RNAi) pathway; promote mRNA degradation	[44]
Y RNA	Y RNA	21–24	Necessary for DNA replication through interactions with chromatin and initiation proteins; target of autoimmune antibodies	[45]
Micro-RNA	miRNA; miR	21–24	Function in RNA silencing and post-transcriptional regulation of gene expression; may have an extracellular localization	[46,47]
Piwi-interacting RNA	piRNA	26–31	Epigenetic and post-transcriptional gene silencing of retrotransposons and other genetic elements in germ line cells	[48]
Small nucleolar RNAs	snoRNAs	60–300	Guide chemical modifications of other RNAs (rRNA, tRNA, snRNA)	[49]
Small nuclear ribonucleic acid	snRNA; U-RNA	≈150	Function in the processing of pre-messenger RNA (hnRNA) in the nucleus; aid in the regulation of transcription factors; telomere maintenance	[50]

**Table 2:** Synopsis of the lncRNA with a role in BC formation. These lncRNA were collected either performing a keyword-based PubMed search or from the database Lnc2Cancer; additional information was retrieved from the original papers or from the GeneCards [58] and OMIM [59] databases.

Name	Acronym	Map	Approx. Length in Kb	Regulation in BC	Notes
Urothelial cancer-associated 1	UCA1	19p13.12	1.4	Up	Cell cycle regulation, cell proliferation, cell migration, cell invasion, apoptosis inhibition
Metastasis-associated lung adenocarcinoma transcript 1	MALAT1	11q13.1	7	Up	Alternative splicing, nuclear organization, modulation of gene expression
Imprinted maternally expressed noncoding transcript H19	H19	11p15.5	6	Up	Modulation of gene expression pre- and post-translation
Taurine Upregulated Gene 1	TUG1	22q12.2	3.3, 5.9, 6.4, 9.7	Up	Cell proliferation and migration
Maternally expressed gene 3	MEG3	14q32.2	1.6 plus other putative sizes	Down	Maternally imprinted gene, chromatin function, angiogenesis, autophagy
MiR-31 host gene	MIR31HG	9p21.3	2.2	Down	Gene expression through DNA methylation, chromatin structure
Long intergenic noncoding RNA upregulated in bladder cancer 1; Bladder Cancer Associated Transcript 1	Linc-UBC1, BLACAT1	1q32.1	3	Up	Cell proliferation, cell motility, invasiveness, colony formation, chromatin structure
LOC572558	LOC572558	9q13	2.6	Down	Cell proliferation, cell motility
Promoter of CDKN1A antisense DNA damage-activated RNA	PANDAR	6p21.2	1.5	Up	Apoptotic response to DNA damage
Gastric carcinoma high expressed transcript 1	GHET1	7q36.1	2.5	Up	Cell proliferation, cell invasion, EMT (epithelial to mesenchymal transition)
Non-coding RNA expressed in aggressive neuroblastoma	ncRAN	17q25.1	2.1, 2.2	Up	Cell proliferation, cell migration, cell invasion, chemotherapy resistance
Growth-arrest-specific transcript 5	GAS5	1q25.1	0.67 (exons only)	Down	Cell division through *CDK6* (cyclin dependent kinase 6) control
Antisense noncoding RNA in the INK4 locus	ANRIL, CDKN2B-AS1	9p21.3	2.7, 3.8	Up	Co-expressed with *CDKN2A* (cyclin dependent kinase inhibitor 2A); cell proliferation and apoptosis
Hypoxia inducible factor 1 alpha antisense RNA-2	HIF1A-AS2, aHIF	14q23.2	1.6	Up	Cell proliferation, cell migration, apoptosis
HOX transcript antisense RNA	HOTAIR, HOXAS	12q13.13	2.2	Up	Regulation of cyclin J via inhibition of miR-205
HOXD antisense growth-associated long non-coding RNA, HOXD cluster antisense RNA 1	HAGLR, HOXD-AS1	2q31.1	15.8	Up	Regulation of tumor size, histological grade and TNM (tumor-node-metastasis) stage
Mediator of DNA damage checkpoint protein 1 antisense RNA 1	MDC1-AS1	6p21.33	N/A	Down	DNA damage checkpoint, apoptosis
Prostate cancer associated transcript 1	PCAT-1	8q24.21	2	Up	Cell proliferation, apoptosis
Plasmacytoma variant translocation 1 (Pvt1) Oncogene	PVT1	8q24.21	N/A	Up	Co-expression with *MYC* (myelocytomatosis viral oncogene homolog), cell proliferation, apoptosis
SWI/SNF complex antagonist associated with prostate cancer 1	SChLAP1	2q31.3	1.7, 1.4, 1.3, 1.1 (major forms)	Up	Cell proliferation, apoptosis, cell migration
SPRY4 intronic transcript 1	SPRY4-IT1	5q31.3	0.7	Up	Cell proliferation, cell migration, cell invasion
ZEB2 Antisense RNA 1, Zeb2 natural antisense transcript	ZEB2-AS1, ZEB2NAT	2q22.3	0.7	Up	TGFβ1 (transforming growth factor beta 1) signaling and epithelial to mesenchymal transition (EMT) control
Ultraconserved RNA 8+, translated ultraconserved region 8+	uc.8+, T-UCR 8+	1p36.22	0.2	Up	Interaction with miR-596; cell invasion, migration, and proliferation
Nuclear enriched abundant transcript 1	NEAT1	11q13.1	3.2	Up	Cell proliferation, miR-101 interaction
Apoptosis associated transcript in bladder cancer	AATBC	21q22.3	4.6	Up	Cell proliferation, apoptosis
LncRNA-n336928	lncRNA-n336928	N/A	N/A	Up	N/A
Up-regulated in non-muscle invasive bladder cancer	lncRNA-UNMIBC	N/A	N/A	Up	Tumor relapse, chromatin structure
Tenascin XA pseudogene	TNXA	6p21.33	4.6	Down	N/A
CADM3 antisense RNA 1	CADM3-AS1, CTA-134P22.2	1q23.2	n/a	Down	N/A
C5orf66 antisense RNA 1	C5orf66-AS1, CTC-276P9.1	5q31.1	1.2	Down	N/A
Keratin 19 pseudogene 3	KRT19P3	4q25	0.9	Up	N/A
AB074278	AB074278	17q23.2-23.3	N/A	Up	Possible functional interactions with TANC2 (tetratricopeptide repeat, ankyrin repeat and coiled-coil containing 2) and EMP1 (epithelial membrane protein 1)

**Table 3:** List of microRNA (miR) with an established role in BC and at least one recognized target gene. miR are ordered according to their first digit, then second digit, and so on; notes are taken from the OMIM database [59] and describe either the molecular or the cellular function of the target gene.

miR	Regulation in BC	Proposed Target Gene	Notes on Target Gene	Refs *
miR-1	Down	SRSF9/SRp30c	Splicing, apoptosis	[17,173]
		TAGLN2	Neuronal protein	[173]
		LASP1	Oncogene	[173]
		PNP	Purine nucleoside phosphorylase	[173]
		PTMA	Hormone polypeptide precursor	[173]
miR-10	Up	RASSF1	Cell cycle inhibitor	[181]
		MAPK1	Cell growth, adhesion, survival, differentiation	[181]
		PKC	Kinase	[181]
		GRB2	Growth factor-induced activation of RAS	[181]
		FGFR3	Cell cycle control; angiogenesis	[181]
		ATM	DNA repair	[181]
		MDM2/4	Ubiquitin ligase targeting TP53	[181]
miR-10b	Up	HOXD10	Homeobox, transcription factor	[173]
		KLF4	Transcription factor	[173]
miR-100	Down	FGFR3	Cell cycle control; angiogenesis	[17]
		MTOR	Protein kinase	[173]
miR-101	Down	PLCG	Actin organization, cell migration	[181]
		FGFR3	Cell cycle control; angiogenesis	[181]
		EZH2	Histone methyltransferase	[185]
		COX2	Inflammation	[173]
		MET	Oncogene, growth factor receptor	[173]
		VEGFC	Growth factor	[173]
miR-103	Up	MSK1	Kinase	[181]
		PKC	Kinase	[181]
		FGFR3	Cell cycle control; angiogenesis	[181]
miR-1182	Down	TERT	Telomerase	[173]
miR-124-3p	Down	ROK1/DDX52	Putative RNA helicase	[173]
		CDK4	Cyclin-dependent kinase	[173]
miR-125	Down	RAF1	Kinase, oncogene	[181]
		KRAS	Activation of mitosis	[181]
		FGFR3	Cell cycle control; angiogenesis	[181]
		CDKN2A	Proliferation	[181]
		TP53	Proliferation, apoptosis, angiogenesis	[181]
miR-125b	Down	E2F3	Transcription factor	[17,173]
		MMP13	Matrix metalloproteinase	[173]
		SPHK1	Sphingosine kinase	[173]
miR-128	Down	VEGFC	RAS regulator, growth factor	[173]
miR-129	Down or up	GALNT1	Post-transcriptional glycosylation	[17,173,185]
		SOX4	Transcription factor	[17,173,185]
		SHC4	Acetylcholine receptor clustering	[181]
		PKC	Kinase	[181]
		GRB2	Growth factor-induced activation of RAS	[181]
		MDM4	Ubiquitin ligase targeting TP53	[181]
		ATM	DNA repair	[181]
miR-133a	Down	EGFR	Cell proliferation, differentiation, motility, survival	[173]
		FSCN1	Actin-bundling	[173]
		GSTP1	Detoxification	[173]
		LASP1	Oncogene	[173]
		PNP	Purine nucleoside phosphorylase	[173]
		PTMA	Hormone polypeptide precursor	[173]
		TAGLN2	Neuronal protein	[173]
		KRT	Keratin	[186]
miR-133b	Down	AKT1	Protein kinase	[173]
		BCL2L2	Apoptosis	[173]
		EGFR	Cell proliferation, differentiation, motility, survival	[173]
		KRT	Keratin	[186]
miR-135a	Down	FOXO1	Cell cycle regulation, apoptosis	[173]
miR-138	Down	ZEB2	Transcription repression	[173]
miR-143	Down or up	ERK5/MAPK7	Kinase	[17,185]
		AKT	Kinase	[17,173,185]
		PDGFB	Growth factor	[181]
		PDGRFB	Inorganic phosphate transporter	[181]
		PKC	Kinase	[181]
		SOS1/2	Positive regulator of RAS	[181]
		KRAS	Activation of mitosis	[181]
		RAF1	Kinase, oncogene	[181]
		ATM	DNA repair	[181]
		TP53	Proliferation, apoptosis, angiogenesis	[181]
		SERPIN	Serine proteinase inhibitors	[173]
miR-144-5p/3p	Down	CCNE1	Cyclin	[173]
		CCNE2	Cyclin	[173]
		CDC25A	Phosphatase, cell cycle	[173]
		PKMYT1	Membrane-associated cdc2-inhibitory kinase	[173]
miR-145	Down	PKC	Kinase	[181]
		FGFR3	Cell cycle control; angiogenesis	[181]
		CBFB	Transcription factor	[173]
		CLINT1	Early and recycling endosomes	[173]
		FSCN1	Actin-bundling	[173]
		ILK	Protein kinase	[173]
		PAK1	Protein kinase	[173]
		PPP3CA	Protein phosphatase	[173]
		SERPIN1	Serine proteinase inhibitors	[173]
		SOCS7	Cell signaling, cytoskeleton	[173]
		IGF1R	Growth factor receptor	[173]
miR-150	Up	PDCD4	Tumor suppressor	[173]
miR-152	Up	DNMT1	DNA methylation	[185]
miR-155	Up	CASP3	Apoptosis	[185]
		TP53BP1	Apoptosis	[185]
		SOCS1	Cytokine response	[185]
		PTEN	Tumor suppressor gene	[185]
		PDCD4	Tumor suppressor	[185]
		SHIP1	Cell differentiation	[185]
		DMTF1	Transcription factor	[173]
miR-16	Down	CCND1	Cyclin	[173]
miR-182-5p	Up	RECK	Tumor suppressor, cell shape	[173,184]
		SMAD4	Signal transduction of the transforming growth Factor-beta	[173,184]
miR-186	Down	HMGN5	Nucleosome, transcription activation	[173]
miR-1826	Down	CTNNB1	Wnt/beta-catenin regulator	[17]
		MEK1	RAS regulator	[17]
		VECFG	RAS regulator, growth factor	[17]
miR-19a	Up	PTEN	Tumor suppressor gene	[173]
miR-193a-3p	Down	LOXL4	Extracellular matrix formation	[173]
		PSEN1	NOTCH receptor cleavage	[173]
		HOXC9	Homeobox, transcription factor	[173]
miR-195	Down	CDK-4	Cyclin-dependent kinase	[17,173]
		RAF1	Kinase, oncogene	[181]
		MAP2K1/2	Kinase, cell growth	[181]
		MAPK1	Cell growth, adhesion, survival, differentiation	[181]
		SOS1/2	Positive regulator of RAS	[181]
		GRB2	Growth factor-induced activation of RAS	[181]
		FGFR3	Cell cycle control; angiogenesis	[181]
		BIRC5	Apoptosis	[173]
		CDC42	GTPase	[173]
		GLUT3	Glucose transporter	[173]
		WNT7A	Cell signaling	[173]
miR-200b/c	Down or up	ERRFI-1	Regulator of EGFR	[17,185]
		ZEB1	Transcriptional repressor	[17,184,185]
		MMP16	Matrix metalloproteinase	[173]
		BMI1	Oncogene	[173]
		E2F3	Transcription factor	[173]
miR-203	Down	BCL2L2	Apoptosis	[173,185]
		BIRC5	Apoptosis	[173,185]
miR-205	Down	TP53	Proliferation, apoptosis, angiogenesis	[17,185]
		PTEN	Tumor suppressor gene	[17]
		C-ERB-B-3	Receptor tyrosine kinase	[17]
		CDC42	GTPase	[17]
		YES	Tyrosine kinase	[17]
		ZEB1/2	Transcription repression	[184]
miR-21	Up	TP53	Proliferation, apoptosis, angiogenesis	[17]
		TIMP3	Degradation of extracellular matrix	[17]
		BCL2	Apoptosis	[17]
		PTEN	Tumor suppressor gene	[17,185]
		TPM1	Tumor suppressor gene	[17]
		MSH2	DNA repair	[17]
		E2F3	Transcription factor	[17]
		ATM	DNA repair	[181]
		VEGFC	Growth factor	[185]
		PDCD4	Tumor suppressor	[185]
		TPM1	Tumor suppressor	[185]
miR-210	Up	VEGF	Growth factor	[185]
miR-214	Down	PDRG1	Oncogene	[173,185]
miR-218	Down	BMI1	Oncogene	[173]
		LASP1	Oncogene	[173]
miR-221	Down or up	TRAIL	Apoptosis	[17]
		ATM	DNA repair	[181]
		MDM2	Ubiquitin ligase targeting TP53	[181]
		STMN1	Microtubule dynamics	[173]
miR-222	Up	PTEN	Tumor suppressor gene	[185]
miR-223	Up	TP53	Proliferation, apoptosis, angiogenesis	[181]
miR-224	Up	SUFU	Tumor suppressor	[185]
miR-23a/b	Up	MAPK1	Cell growth, adhesion, survival, differentiation	[181]
		FGFR3	Cell cycle control; angiogenesis	[181]
		EGFR	Cell proliferation, differentiation, motility, survival	[173]
		MET	Oncogene, growth factor receptor	[173]
		ZEB1	Transcription factor	[173,184]
miR-24	Down	CARD10	Apoptosis	[173]
		FOXM1	Transcription factor	[173]
miR-26a	Down	HMGA1	Non-histone chromosomal protein	[185]
		PLOD2	Collagen crosslinking enzyme	[187]
miR-27a	Down	SLC7A11	Cystine/glutamate exchanger	[173,185]
		RUNX1	Transcription factor	[173]
miR-27b	Down	DROSHA	miR biogenesis	[173]
		EGFR	Cell proliferation, differentiation, motility, survival	[173]
		MET	Oncogene, growth factor receptor	[173]
miR-27a-3p	Down	EGFR	Cell proliferation, differentiation, motility, survival	[185]
miR-29a/b/c	Down or up	PDGFRA	Growth factor	[181]
		FGFR3	Cell cycle control; angiogenesis	[181]
		MCL1	Apoptosis	[185]
		DNMT3A/B	DNA methyltransferase	[185]
		CDK6	Cyclin-dependent protein kinase	[173]
miR-30a/c	Down	PLCG	Actin organization, cell migration	[181]
		MAPK1	Cell growth, adhesion, survival, differentiation	[181]
		PDGFA	Growth factor	[181]
		ATM	DNA repair	[181]
		TP53	Proliferation, apoptosis, angiogenesis	[181]
		NOTCH1	Cell signaling	[173]
miR-31	Down	FGFR3	Cell cycle control; angiogenesis	[17,185]
miR-320a/c	Down	ITGB3	Cell adhesion	[173]
		CDK6	Cyclin-dependent protein kinase	[173]
miR-34a	Down	NOTCH1	Cell signaling	[173,185]
		CD44	Cell adhesion	[173]
		HNF4G	Nuclear receptor	[173]
miR-424	Down	EGFR	Cell proliferation, differentiation, motility, survival	[185]
mir-449a	Down	CDK6	RB control	[17]
		CDC25a	RB control	[17,173]
		TP130	RB control	[17]
miR-485-5p	Down	HMGA2	Non-histone chromosomal proteins	[173]
miR-490-5p	Down	FOS	Oncogene, transcription factor	[173]
miR-493	Down	FZD4	Transmembrane receptor	[17,173]
		RhoC	G protein	[17,173]
miR-497	Down	BIRC5	Apoptosis	[173]
		WNT7A	Cell signaling	[173]
miR-574-3p	Down	MESDC1	Mesoderm development	[173]
miR-576-3p	Down	CCND1	Cyclin	[173]
miR-590-3p	Down	TFAM	Mitochondrial transcription factor	[173]
miR-7	Down	SHC1	Cell signaling	[181]
		PKC	Kinase	[181]
		HOXB5	Homeobox, transcription factor	[184]
miR-708	Up	CASP2	Apoptosis	[173]
miR-9	Up	CDH-1	Cell differentiation	[185]
		CBX7	Chromatin structure, transcription repression	[173]
		CERS2	Cell signaling	[173]
miR-96	Down	CDKN1A	Proliferation	[173]
miR-99a	Down	FGFR3	Cell cycle control; angiogenesis	[17,173]

* To avoid a long list of articles, References (Refs) refer to recent and comprehensive reviews and one scientific article about miR and BC. The reader can find more information in the shown bibliography. miR are reported using the names found in the references and related original articles, thus reflecting different types of nomenclature used in this field.
